# On the Effects of Blisters on the Young Subject

**Published:** 1847-09

**Authors:** 


*On the effects of Blisters on the Young Subject.* By John B. Beck, M. D., Professor of Materia Medica and Medical Jurisprudence in the College of Physicians and Surgeons of New York.

It has frequently struck me that a treatise, describing with the necessary precision, the peculiarities of the effects of medicinal agents on the young subject, as distinguished from their effects on the adult, has long been needed in our profession. As yet I know of no such work. The systems of Materia Medica, valuable and elaborate as they are, and in which we should naturally look for the requisite information, are confessedly deficient on this subject. The consequence is that the young practitioner who depends upon them, finds himself continually embarrassed in the treatment of the diseases of children, and he is obliged after all, to rely upon the incidental observations gathered from works on general practice, or upon the slow accumulation of his own observations. Even works professedly on the diseases of children, do not supply the want. They indeed specify doses suitable to the age, and now and then give cautions in relation to the use of certain medicines, but they do not enter into the philosophy of the subject as it ought to be engaged upon. It is treated by them more as a matter of enlightened empiricism, than as one founded on sound and rational physiological and pathological principles. In some previous papers, I have endeavored to offer some contributions on this subject, and should they be the means of inducing some experienced hand properly to elaborate it, it appears to me that a greater practical benefit could not be conferred on the profession. On the present occasion, I propose, to make *Blisters* the subject of a few remarks.

The first peculiarity attending the operation of blisters on the young subject is, *that they produce their effects in a shorter time than they do in the adult.* This a fact well known to every practitioner. While in the adult, they do not produce their effects until from eight to twelve or even more hours have elapsed, in the child the same takes place in from two to six hours. In this respect there is a striking difference between blisters and most other remedies. Emetics and cathartics, for example, do not appear to act with any more rapidity on the child than they do on the adult. Now this fact, of the more prompt action of this class of agents, upon the child, although a simple one, is nevertheless one of great importance, and one which should be continually borne in mind. It has a practical bearing, not merely upon the mode of conducting the process of blistering in young subjects, but also upon the use of it in their various diseases.

The second peculiarity is, *that the local inflammation produced by a blister is greater in the young subject than in the adult.* The reason of this is obvious. In infancy, the skin is more delicate in structure, has greater vascularity, and a higher degree of sensibility; all circumstances favoring the development of greater inflmmation. The local impression, accordingly, made by a blister, is not merely more rapidly developed in the young subject, but it is also more intense.

The third peculiarity is that in young subjects *blisters are more apt to be followed by the injurious consequences of inflammation, such as ulceration, gangrene, and even death.* Numerous and melancholy instances of this are to be found on record. Dr. Ryan, speaking of the use of blisters in children, says, "I have seen a blister on the chest followed by sloughing, and an aperture form over the epigastrium, which exposed the subjacent viscera."* Dr. Thompson states, that he "has seen gangrene and death follow the application of a blister on an infant."† Dr. North states that he has "twice known infants destroyed in consequence of the sloughing of blisters, the progress of which could not be arrested."‡ Professor Chapman remarks, that in children a blister "sometimes induces gangrene, as I have witnessed in two or three instances."§ My friend Dr. W. C. Roberts informs me, that he has met with two cases in which children sank under the effects of blisters. Numerous other facts of a similar character might be adduced to show the disastrous effects which sometimes result from the application of blisters to children; and to the minds of many physicians it constitutes a serious objection to their use in their diseases. Dr.

* Manual of Midwifery, &c. By Micheal Ryan, M. D. p. 476.

† Materia Medica. By Anthony Todd Thompson, M. D. Vol. II., p. 535.

‡ Practical Observations on the Convulsions of Infants. By John North, p. 202

§ Elements of Therapeutics, &c. Vol. II., p. 28

Armstrong says, "I have a great dread of the application of blisters to infants, on account of what is called the local and constitutional irritation."* Now these occurrences may and do take place also in the adult, but they are comparatively rare, and only under very peculiar conditions of the system. In infants on the contrary, they are by no means uncommon. In any child, however healthy, they may occur from the simple cause of their being left on too long. They are more likely to take place however, in certain conditions of the system or of the skin itself. Thus, for example, in cases where a child is greatly emaciated, or the constitution broken down from various causes, the inflammation of a blister is very apt to become unhealthy in its character, and to be followed by injurious consequences. Then again, where the skin itself is in a diseased state, it is much more likely to happen than in the healthy conditions of that surface.

The fourth peculiarity is, *that the constitutional excitement produced by blisters is generally greater in young subjects than in the adult.* That this must necessarily be so obvious. In all cases, the general excitement must be in proportion to the degree of local irritation and the sensibility of the patient's system. If so, the general vascular and nervous excitement produced in the child by a blister, must, as a matter of course, be greater than in the adult. So powerful indeed is the impression thus made sometimes, that convulsions have been produced from this cause. Dr. North says: "I have freequently seen very severe paroxysms (of convulsions) brought on in consequence of their injudicious and unnecessary application."†

From the whole of the foregoing, it is evident that blisters are much more powerful in their agency upon the young subject than upon the adult. They operate with more rapidity—cause a greater degree of local irritation and constitutional excitement—and their operation is frequently followed by consequences which rarely occur in the adult.

If such be the case, it appears to me that some conclusions may be drawn of no inconsiderable practical importance.

1. If blisters are more powerful in their action upon children than adults, then it would seem to follow that they may be rendered more efficient as a means of cure in their diseases. And such appears to me to be really the fact. In all cases, where their revulsive agency is required, and where they are properly applied, it has struck me, that more decided benefit has resulted from their use in children than in adults, and that too, under circumstances as nearly similar as they well could be. Besides acting more powerfully the rapidity of their operation in children, gives them a great advantage in many cases. We all know that one of the great

* Lectures, p. 362.

† Observations on the Convulsions of Infants. By J. North, p. 209.

objections to a blister in the adult, sometimes at least, is the length of time which it takes to produce its effects. In a child this is in a great measure obviated, we have in a blister not merely a powerful but a comparatively speedy counterirritant. As remedial agents therefore, in the diseases of children, it seems to me that they ought to hold a high rank. I am aware, that by some an opinion, entirely the reverse of this is entertained. Mr. North, in his valuable work on the Convulsions of Infants, that he thinks, that except as stimulants, in depressed states of the system, blisters are altogether objectionable in the diseases of children. As revulsives in cases of local inflammation, he regards them as having gained a character which they do not merit, and that in fact they do more harm than good. On this subject he says, " the period at which we apply blisters in local inflammatory affections is not to be forgotten. We first subdue the severity of the disease by other and appropriate remedies, and when it is upon its decline, when in all probability the unassisted powers of nature would successfully perform the remainder of the task, a blister is applied. The patient gets well, notwithstanding the additional pain thus inflicted; and the fortunate result of the case, which is really to be attributed to the measures previously employed, is said to be owing to the good effects of counterirritation, &c., and the blister gains a character, to which in point of fact it has no claim."* Now all this may no doubt be true in some cases, but that it is so generally, can hardly be admitted. It should be recollected, that in the treatment of local inflammations, blisters are only auxiliary remedies, of themselves, and alone, capable of doing but little, and yet when cooperating with other agents, such as bloodletting. &c., exceedingly powerful and valuable Every one knows that there are periods and conditions in the career of inflammatory complaints, when bleeding and other reducing remedies have been carried to the fullest extent deemed advisable, and yet sufficient disease may remain, if not to destroy life,yet to render convalescence tedious, or to lay the foundation of subsequent chronic disease. This of course it is all important to obviate. Now it is just under this condition of things that blisters come in with great effect, and frequently break up completely the remaining vestiges of disease, and in this way I look upon them as remedies, acting with more power and efficiency in children even than in adults.

2. From the fact of blisters being such powerful agents, and especially from the fact of their being so liable to be followed by dangerous consequences, more caution is required in their use in children than in adults. Important and valuable as they are and may be made, if properly used, their indiscriminate application cannot be too much reprobated. Just in proportion to the good they are capable of accomplishing under proper circumstances, is the

* Observations on the Convulsions of Infants. By John North, p 205—6.

evil which results from them, if heedlessly or injudiciously resorted to. It is to be feared that this is not always borne in mind as it should be. As a general rule, they should never be resorted to, especially in very young children, unless some decided benefit is anticipated from them.

3. The mode of conducting the process of blistering in a young subject is a matter of great nicety, and should call for the utmost care on the part of the practitioner. As one of the principal causes of gangrene, is the leaving the blister on too long, this is a point which should be specially attended to. To many this may appear a small matter, but it is really one of great moment, and in relation to which I am sorry to say that the directions given in many of our practical works are so discordant, as to be very poor, if any, guides to the young practitioner. By way of illustration, I will quote a few of them. Dr. Armstrong says, "from twelve to sixteen hours is generally sufficient for the application of the blister in adults, and half that period in children."* Dr. Williams says, that "to avoid gangrene in children, it is advisable never to allow the blister to remain on more than six hours."† Dr. Dewees states that "in children, the blister is frequently found to have performed its duty in eight hours, and very often in six. It should therefore, always be examined at these periods, and dressed if sufficiently drawn; if not, it should be suffered to remain until this take place."‡ Evanson and Maunsell say, "in no instance is the blister to be left on more than a few hours (from two to four)—not longer, in fact, than until the skin is reddened, when vesication will follow; but this result should not be waited for, as attendants always will do, unless the most express directions to the contrary be given."§ Neligan directs that "as a general rule, in infants and young children, blisters should only be left on until redness of the surface is produced, when the application of a warm poultice to the part will cause vesication."|| Ballard and Garrod remark, that in children a blister should not be allowed to remain on longer than to produce redness of the surface; and they add, "in very young infants, it has appeared to us doubtful whether even redness should be permitted to occur before its removal."¶ The foregoing is a sample of the discrepancy of opinion in relation to a most important point of practice, and one confessedly too, not unfrequently involving the life of the young subject, as advanced by authors of the highest respectability, and who may be supposed to exert a wide influence

---

* Lectures, &c. By John Armstrong, M. D., p. 3[illegible]2.

† Cyclopœdia of Practical Medicine. American Edition. Vol. I., p. 529. Art. Counter-irritation.

‡ Practice of Physic. By Wm. P. Dewees, M. D., p. 28.

§ A Practical Treatise on the Management and Diseases of Children. By R. T. Evanson, M. D., and H. Maunsel, M. D., p. 107.

|| Medicines, their uses and mode of administration. By J. W. Neligan. M. D., p. 202.

¶ Elements of Materia Medica and Therapeutics. By Ed. Ballard, M. D., and A, B. Garrod, M. D., p. 457.

in guiding the practice of young beginners in our profession. The fact is, and this perhaps may account somewhat for the difference of opinion just noticed, that no positive rule can be laid down in relation to the precise time that a blister should be left on a young child. From the original differences in the sensibility of the skin in children, the period must necessarily vary, and the only safe general rule, is to be governed by the actual effect produced. For this purpose the blistering plaster should be raised at suitable intervals and the state of the skin observed. And the safe plan is, according to the directions of some of the authors quoted above, to remove the blister as soon as the surface appears uniformly red, and then to apply a soft poultice. In most cases this will be followed by suitable vesication, while any injurious consequences will be averted.

It is not my intention in this paper to go into the minutiæ of conducting the process of blistering, but there is one other point which I cannot help noticing, and that is, the practice which is so common with some of covering the blistering plaster with dry fly-powder. Although intended to make the blister more potent, it frequently has a directly contrary effect, from the fact that the blister does not adhere so closely to the skin; over and over again have I seen blisters prepared in this way fail in producing the desired effect, although left on even longer than the usual period. Then again, the dry powder is apt to adhere to the skin after the blister is removed, and in this way strangury is more likely to be producad. In one case, according to Ure, sphacelus has occurred from this cause.* As apothecaries are very apt to prepare blisters in this way, it is important that practitioners should be on their guard to prevent it. With regard to the dressing of a blister, always a matter of importance to the young subject, and frequently so to the adult, I would call the attention of the reader to a mode very recently recommended by Dr. D. Maclagan, of Scotland, which holds out many advantages over the ordinary method. After leaving the blister on for a suitable time, he applies a poultice of bread and milk for two hours. After discharging the serum, a thick layer of soft cotton wadding is applied over the part, with the undressed or wooly surface next the skin. If in the course of a few hours this should become soaked with the serous discharge from the blister, so much of the cotton may be removed as can be done without disturbing the loose epidermis beneath, and the whole again covered with a dry layer of cotton. This is all the dressing which in general is requisite. The cotton is allowed to stick to the skin of the blistered part, and when a fresh layer of epidermis is formed, which takes place very readily, the old epidermis and cotton come off together, leaving a smooth whole surface below.

* A Practical Compendium of the Materia Medica, &c. By Alexander Ure, M. D. p. 31.

The advantages of the above mode, according to Dr. M., are first, "that it renders the blister much less painful and annoying to the patient than when unguents are used. The tenderness in fact is comparatively so trifling, and the protection by the cotton so good," he says, "that I have been enabled without annoyance to the patient to percuss freely, and apply the stethoscope firmly over the blistered parts, which had been dressed for the first time only an hour or two previously; secondly the blister heals faster under it than under dressings with cerate: for although the cotton may remain adhering for some days, I have generally found, that within twelve hours the patient ceases to feel the blister a source of annoyance. Lastly, it dispenses with the greasy applications so disagreeable to patients of cleanly habits."*

4. To obtain the good and avoid the evils of blisters, it is evident that a nicer discrimination of the conditions of the system is necessary in the use of this class of agents in children than in adults. Long experience has established the fact that it is only under certain states of the system, that blisters can be used with any prospect of advantage. If this be true in the adult, it is doubly so in the young subject, and any mistake in this respect is much more likely to be followed by injurious consequences in the latter than in the former. Now the conditions which influence the effects of these agents, are the state of the skin, and the state of the nervous and vascular systems. With regard to the skin, it may be laid down as a general rule, that when blisters are used as revulsives, the part to which they are applied should be as nearly as possible in a state of perfect health. In this state, the irritation of blistering may be established even in a child with comparative safety. On the contrary, when the skin is in a morbid state, ulceration and gangrene are by no means unusual occurrences. All this is occasionally illustrated in scarlatina and measles. Mr. Pereira mentions that he has seen "two instances of death from the gangrene caused by applying a blister after measles."† My friend Prof. Dunglison, in his valuable work on Materia Medica, states that he has seen "several cases of death manifestly caused by the use of blisters in scarlatina and measles."‡ Other facts of a similar character might be adduced, but the preceding are sufficient to show the tendency which there exists in this state of the skin to take on unhealthy inflammation. And the reason is to be sought for in the changed condition of the skin. During the febrile stage of these diseases the skin is preternaturally injected and excited. As soon as the fever subsides and the eruption recedes, the skin is left in a state of debility—a state in which, as we all know, inflammation is very likely to terminate unfavorably. I hope it may not be inferred from the preceding, that I mean to express the opinion

* Edinburg Monthly Journal of Medical Science, May 1847, p. 834.

† Materia Medica, Vol. II., p. 775. American Edition.

‡ Vol. II., p. 219.

that blisters ought never to be used in such cases as measles and scarlatina—but the possible occurrence of such consequences ought to make us exceedingly cautious about the manner of using them, and indeed ought to deter us from using them at all, unless under a manifest necessity. In every case, therefore, before applying blisters to young children, the condition of the skin ought to be attended to.

With regard to the state of the system, this is even still more necessary to be inquired into. Indeed this is all important, if we hope to realize any of the expected benefits from these agents. Now there are two states of the system almost equally unpropitious to their use—and these just the reverse of each other. The first is that in which high inflammatory excitement is present. That this is unfavorable to the legitimate operation of a blister as a revulsive, is obvious, if we reflect for a moment upon the effects of this agent. These are, local irritation and general excitement. Now in all cases where an internal inflammation exists, the difficulty of resolving it by any means will be proportioned to the degree of general excitement accompanying it. If a blister be applied where this general excitement is already very great, one of the necessary consequences will be to augment this so greatly as to counteract, in a greater or less degree, according to circumstances, the beneficial effects of the blister as a revulsive. Under this condition of things, the internal inflammation will be aggravated instead of abated, in consequence of the increase of general excitement. Hence the fact has been generally observed, that if blisters are applied in the early periods of inflammatory complaints, or before suitable evacuations have been resorted to, they frequently do more harm than good. They merely add fuel to the fire.

On the other hand, a state of great constitutional exhaustion and emaciation is also unfavorable to their operation. The reason here, however, is entirely different from that in the preceding case. The danger here is that from the impaired state of the vital energies, the local inflammation of the blister may be followed by ulceration, gangrene and death. In the use of blisters, therefore, both these extremes should be carefully avoided. With regard to the condition most propitious to their use, it is that in which the general excitement is rather below than above the natural standard. When this is the case, there is less danger from any increase of excitement, while the system is in the state most favorable to the transfer of irritations from one part to another. Now all this is applicable to the adult, and we can easily see how much more so it must be the case of the irritable and sensitive infant.

5. In the use of blisters in children, especial reference should be had to the peculiarities of their temperament and constitution. This is more important perhaps than it may at first sight appear. Every practitioner must have observed the extreme suffering which adults sometimes undergo from the irritation of blisters. In nervous and irritable habits I have myself seen a state of things thus induced, little short of phrenzy. In children of nervous temperaments all this is much more likely to happen, and accordingly greater caution should be exercised.

If the foregoing conclusions be founded in truth, they would seem at once to expose the impropriety of the practice of resorting to the use of blisters on every trifling occasion, in the management of the diseases of children. There is an opinion prevalent—how it is originated I know not, that blisters are innocent remedies—if they do no good, they can do no harm. Now this is unquestionably a great error, and has been productive of vast mischief. Independently of the unnecessary suffering which they may occasion, they sometimes produce death by the manifest causes of ulceration and gangrene, while in others they insidiously aggravate the disease they were intended to relieve.—*New-York Journal of Medicine, and the Collateral Sciences.*

